# Lifestyle behaviour and prevalence of cardiovascular risk factors - a pilot study comparing Kiribati and European seafarers

**DOI:** 10.1186/s12889-019-7186-2

**Published:** 2019-07-01

**Authors:** R. von Katzler, B. C. Zyriax, B. Jagemann, J. Westenhoefer, H. J. Jensen, V. Harth, M. Oldenburg

**Affiliations:** 1 Institute for Occupational and Maritime Medicine (ZfAM) Hamburg, Seewartenstr, 10, 20459 Hamburg, Germany; 2 Preventive Medicine and Nutrition, Institute for Health Services Research in Dermatology and Nursing (IVDP), University Medical Center Hamburg-Eppendorf, Hamburg, Germany; 3 Medical Clinic and Polyclinic, University Medical Center Hamburg-Eppendorf, Hamburg, Germany; 4 Competence Center Health, Faculty of Life Sciences, Hamburg University of Applied Sciences, Hamburg, Germany

**Keywords:** Seafarers, Cardiovascular, Lifestyle, Kiribati, Obesity, Vessels

## Abstract

**Background:**

According to internal observations within a German shipping company, obvious risk-behaviour persists among the crew members coming from the Pacific Island State of Kiribati and representing a large part of the crew aboard merchant vessels of this company. These observations were related to excessive eating habits. This study aims to assess the cardiovascular risk among seafarers and to compare lifestyle factors between Kiribati and European crew members.

**Methods:**

In the present maritime field study 81 sailors (48 Kiribati, 33 European, average age at 38.9 and 36.8 years respectively) were examined from April until August 2014 aboard four container ships crossing the Atlantic Ocean (participation rate of 90.9%).

**Results:**

Based on the number of established risk factors, 35.4% of the Kiribati and 16.7% of the European crew members were regarded as a high risk group for cardiovascular diseases. The HDL-values of Kiribati were found to be considerably lower (34.9 mg/dl) than the references values given by the WHO and in comparison to the European crew members (44.8 mg/dl) (*p* = 0.002). 91.7% of Kiribati and 51.5% of European participants were found to be overweight according to WHO-criteria - with a mean Body Mass Index (BMI) of 30.3 kg/m^2^ and 25.6 kg/m^2^ (*p* <  0.001). Regarding lifestyle factors Kiribati often claimed to eat significantly larger amounts of food aboard while most European sailors stated to eat less or about the same during their shipboard stay (*p* = 0.017). Daily sleeping hours were slight on both sides; however with a mean of 5.2 h a day Kiribati crew members had significant fewer sleep (*p* = 0.038). The examined Kiribati sailors had a mean increase in weight of 6 kg over a 12 months period of observation.

**Conclusions:**

In total the compiled data points towards a higher risk of cardiovascular diseases particularly due to alimentary habits within the Kiribati crew members. The distinct weight-gain measured among the Kiribati in spite of higher energy consumption levels at sea is alarming. Thus, the results of this study confirm the necessity of health-improving interventions aboard cargo vessels.

## Background

Obesity is one of the well-known risk factors for cardiovascular diseases. Over the last years, research in Poland, France, Norway and Germany has repeatedly shown that cardiovascular risk factors like obesity, smoking, non-HDL-cholesterol, lack of movement and unfavourable eating habits are often prevalent among sailors [1–5].

Literature on cardiovascular risk factors among seafarers is abundant, however the focus often only lies on European sailors. The review by Pougnet et al. (2013), which comprises 18 publications on the subject of cardiovascular risk factors in sailors, included a total of 57,473 subjects, of which only 327 were of non-European origin [6].

Crew members staying aboard cargo vessels for several months at a stretch have very little influence on the quality and diversity of their nutrition. The rare opportunities for shore leave hinder them in buying their own more diverse grocery supplies. Hence, seafarers’ nutrition depends mainly on the food provided on board, which has often been described as a high-fat diet. Babicz-Zielinska and Zabrocki (1998) [7] as well as Lawrie et al. (2004) [8] observed that the main dishes aboard cargo ships mostly consist of meat. Conditions aboard, such as diverse eating habits of a mostly multinational crew, the division into officer and crew mess rooms as well as the constantly changing work schedules increase the difficulties of regulating a healthy diet [9], a circadian rhythm and regular physical activities even more. Although balanced nutrition at sea has become a bigger issue in the public discussion, there are still very few in-depth studies on this matter [10].

From a medical point of view, the population of Kiribati features characteristics specific to the Pacific island state [11, 12]. In 2015, 39.3% of the male Kiribati population was classified as obese [13]. In comparison, according to OECD (2012) [14], 16.5% of the European population was considered as obese. The past decades have shown a trend towards adiposity in many developing countries. Imported nutritional goods, such as canned and pre-packaged convenience foods, are a crucial reason for this development. Studies have shown that the diet of the Kiribati is characterised by an extremely low intake of essential omega-6 fatty acid, whereas the consumption of saturated fatty acids is excessively high [15].

In the last couple of years, the shipping company allocating the four cargo vessels for this study received reports about impairments of health due to cardiovascular symptoms while at sea. This seems to be a crucial problem, particularly among the Kiribati crew members. The question of whether these health restrictions could be caused by malnutrition and the observed weight gain during the time on board was discussed.

This study aims at analysing the cardiovascular health status of crew members at sea, especially with respect to lifestyle-related risk factors and in a comparison between Kiribati and European sailors.

## Methods

The 81 seafarers participating in this study came from four cargo vessels belonging to a shipping company with numerous Kiribati crew members. All explored ships were container ships of similar size (mean 99,400 gross tonnage) on a transatlantic route. The participation rate for this study was 90.9% (five women, four Africans and one person of Asian origin were excluded from the analysis due to statistical reasons). This resulted in two homogenous male cohorts: 48 participants with a Kiribati origin and 33 Europeans. Participation in this study was voluntary (participation rate 90.9%) and all personal data were pseudonymised. Participants gave their informed consent and the Ethics Committee of the Hamburg Medical Association approved this study. The study was conducted according to good clinical practice based on the Helsinki declaration. The research is also registered within the German Registry of Clinical Studies under the DRKS-ID: DRKS00010819.

### Medical examination of sailors

In terms of a maritime field study, a physician examined and analysed the above-mentioned seafarers in the course of more than 100 days at sea.

#### Survey questionnaire

The sailors were asked about their demographic, lifestyle and occupational data. Furthermore, the Perceived Stress Scale used allowed for an assessment of their subjective stress level. Various studies have shown that higher values of this scale correlate with an increased risk of certain diseases [16, 17]. There are no explicit cut-off values for analysis of the Perceived Stress Scale. According to a prior large population study (*n* = 2387) [18], participants with values of 20 points and above were regarded in this study to have an elevated stress level.

#### Anthropometrics

The determination of anthropometric values (height and weight) aboard the vessels took place solely during the ships’ stay in port to ensure that movements of the ships could not impact the measured values. Weight measurements were performed with a calibrated Kern® scale on participants wearing lightweight clothing and in a sober state. Waist circumference was measured at the midpoint between the bottom of the ribs (lower costal margin) and the top of the hip bone (iliac crest), while the participant was in the standing position during a normal breath out using a non-stretch tape. To assess weight development among the Kiribati crew members up to the current shipboard examination, information about weight and height was obtained from their prior medical fitness tests ashore. The determined weight changes mainly refer to the duration of stay on board the current vessels.

#### Blood pressure measurements

Blood pressure was measured with acouophonia, taking measurements on each sailor’s upper arm with a pressure sleeve from Boso®. All subjects underwent at least three measurements per day (morning, midday and evening) in a seated position. The average of all individual measurements was calculated for the analysis.

#### Measuring blood parameters

Venepuncture was carried out among all subjects in a fasting state (min. 10 h) in the morning of various days. After a 30-min storage period, the filled blood tubes (S-Monovette® serum gel by Sarstedt®) were centrifuged for 10 min at 2000 x g in a Sarsted® LC-6 centrifuge and immediately stored at − 18° Celsius in a lightproof storage unit.

After completion of the voyage and in compliance with the cold chain, the following blood parameters were determined in a German laboratory (Lademannbogen GmbH, Hamburg, Germany): cholesterol, HDL, non-HDL-cholesterol, LDL, LDL/HDL ratio, triglyceride, alkaline phosphatase, uric acid and fasting glucose. The analysis of all results referred to reference values from German population studies as no reference values were available for Kiribati in the literature.

### Assessment of cardiovascular risks

A number of parameters are available for assessing population-based cardiovascular risk scores. However, several studies have shown that the calculation of a 10-year probability of a heart attack is very imprecise when applied to a group comprising different nationalities [19].

Although the internationally well-known cardiovascular risk factors appear to have a somewhat different relevance within various populations, it can be assumed that they form a valuable basis for the estimation of the general cardiovascular risk [20, 21]. For this risk assessment, 8 internationally established risk factors were used for the entire group (age ≥ 45 years, LDL cholesterol ≥160 mg/dl, active smokers, HDL cholesterol ≤40 mg/dl, blood pressure ≥ 140/90 mmHg, familiar predispositions, fasting blood glucose levels ≥110 mg/dl and triglyceride ≥150 mg/dl) according to the National Cholesterol Education Program III (NCEP 2001) [22]. Corresponding to the study by Oldenburg et al. (2008) [23], in the present study one point was equally assigned to each factor. Therefore, values from 0 to 8 could be obtained by summation of the risk factors (RF). The participants were grouped into categories of either low (< 3 RF) or high (≥ 3 RF) cardiovascular risk according to this individual rating system. This type of risk evaluation is not as specific as the application of risk scores for a certain population. However, it provides a suitable possibility to compare two culturally different collectives.

### Endurance performance capacity

The Chester Step Test was conducted in order to ascertain the seafarers’ endurance performance capacity. This test correlates highly with the medical gold standard of ergospirometry (*r* = 0.92; *p* <  0.001) [24]. It is a multi-level submaximal exercise test used to assess age-related physical fitness. This test estimates the individual oxygen uptake rate (VO_2_) in ml per kg bodyweight per minute according to well-evaluated mathematical equations [25, 26]. In compliance with the Chester Step Test specifications, one of the four possible step heights was chosen prior to the exercise as a suitable individual stress level. A sternum strap (T37) by Polar® was applied to monitor the participants’ heart rates continuously.

### Activity monitoring

The seafarers’ physical activity was examined with the Bodymedia SenseWear® armband monitor carried on the left upper arm. Each participating sailor wore this monitor for roughly 200 h. Its internal sensors include an accelerometer, a thermal flow sensor, a galvanic sensor that records skin response, a skin temperature sensor, and an air temperature sensor. The accelerometer in the armband has two axes and uses a microelectromechanical sensor that measures movement. The software created by the manufacturer calculates energy expenditure using a patented algorithm that combines acceleration, heat flow, and other parameters. This device provided information on the total energy expenditure, the active energy expenditure, daily number of steps and duration of sleep periods [27]. Simultaneously, the subjects kept records on their current activities (working, leisure and sleeping time) in daily protocols. By means of these records, cumulative measurement values of the armband monitor could be assigned to certain activities on a daily basis.

### Statistics

The statistical analysis was conducted with the statistics program SPSS 20 (IBM Corporation). A comparison of the two groups (Kiribati vs European) was performed with the Mann-Whitney U Test. Significant differences in frequency between the two groups were analysed through the Chi-Square Test or, in the case of a smaller number of incidents, with the Exact Test according to Fisher. The level of significance was ≤0.05.

## Results

### Study population

Two homogenous male cohorts were examined: 48 participants with a Kiribati origin and 33 Europeans (age range of the Kiribati: 23–64 years; European: 20–61 years). The average age of the Kiribati (38.9 years) was insignificantly higher than that of the Europeans (36.8 years).

### Anthropometrics

Even though the Kiribati crew members had a smaller average body height, their median BMI was significantly higher than that of European participants (30.4 (SD ± 4.2) kg/m^2^ vs 25.6 (SD ± 3.4) kg/m^2^) (*p* < 0.019). According to classifications by the WHO, 91.7% of the Kiribati and 51.5% of the control group were overweight (BMI ≥ 25 kg/m^2^). While nearly half of the Kiribati groups were classified as obese (BMI ≥ 30 kg/m^2^), only 9.1% of the Europeans were assigned to this classification. Two Kiribati had a BMI above 40 kg/m^2^, which correlates with obesity class 3. The mean waist circumference of the Kiribati was also significantly larger than that of the Europeans (97.5 cm vs. 92.2 cm; *p* = 0.045).

**Fig. 1:**
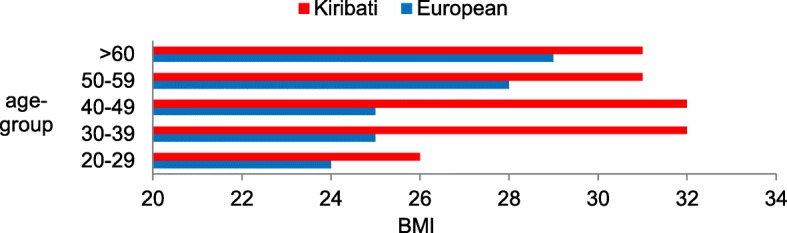
Age-group related Body Mass Index in Kiribati (*n* = 48) and European (*n* = 33) seafarers

As shown in Fig. 1, the average Europeans’ BMI grew constantly with increasing age. In contrast, the Kiribati demonstrated a significant gain in weight among seafarers older than 30 years of age compared to the younger ones. The youngest age group had an almost normal weight median according to WHO criteria, with an average BMI of 26 kg/m^2^. All other age groups of Kiribati showed an average BMI ≥ 30, indicating obesity.

The weight development within the past year was calculated using the information about the prior weight of the Kiribati crew members during their previous medical fitness tests for nautical services. This analysis showed a distinct increase in body weight in correlation with the duration of their stay on board. The stay on board over 6 and 12 months was associated with an average increase in weight of 4.8 kg and 5.9 kg.

Blood pressure measurements demonstrated arterial hypertension among 8% of the Kiribati and 3% of the European sailors.

### Laboratory results

The results of the lipid profile displayed a similar level of total cholesterol in both groups, however the composition differed (Table 1). The Kiribati showed a significantly lower level of HDL-cholesterol with a calculated median below the recommendations by the DGFF (German Society for Combating Dyslipidemia and its Associated Illnesses; > 40 mg/dl). In conclusion, these results revealed significant differences in the non-HDL cholesterol composition.

**Table 1:** Blood parameters in Kiribati and European seafarers

	Kiribati (*n* = 48)	European (*n* = 29)	*p* ^*1*^
Lipid profile			
*Cholesterol (mg/dl) (SD)*	159.9 (39.8)	157.6 (39.7)	n.s.
*HDL (mg/dl) (SD)*	34.9 (10.4)	44.8 (13.8)	0.002
*Cholesterol/HDL (SD)*	4.8 (1.4)	3.8 (1.5)	0.001
*non-HDL cholesterol*	125 (29,4)	112,8 (25,9)	0.033
*LDL (mg/dl) (SD)*	102.3 (31.7)	91.5 (28.3)	n.s.
*LDL/HDL (SD)*	3.1 (1.1)	2.2 (0.9)	< 0.001
*Triglyceride (mg/dl) (SD)*	114.3 (53.9)	110.4 (22.1)	n.s.
Other			
Alkaline phosphatase (U/l) (SD)	63.4 (20.2)	54.1 (14.8)	0.031
Uric acid (mg/dl) (SD)	5.5 (1.2)	4.8 (1.3)	0.025
Fasting blood glucose (mg/dl) (SD)	83.2 (15.6)	81.0 (16.1)	n.s.

^1^Mann-Whitney U Test

The uric acid values in the Kiribati were significantly higher than among the Europeans, while there was no difference between both groups concerning fasting blood glucose levels.

### Cardiovascular risk

The cardiovascular risk could be assessed in 78 participating seafarers. According to the above mentioned procedure, a total of 56 sailors (71.8%) were grouped into a low-risk group (< 3 RF), while 22 subjects (28.2%) were assigned to the group with a high cardiovascular risk (≥ 3 RF). Compared to the Europeans, the Kiribati were considerably more often allocated to the high risk group (35.4% vs. 16.7%). This difference was slightly attenuated after adjustment for age (22.9% vs. 13.3%).

**Fig. 2:**
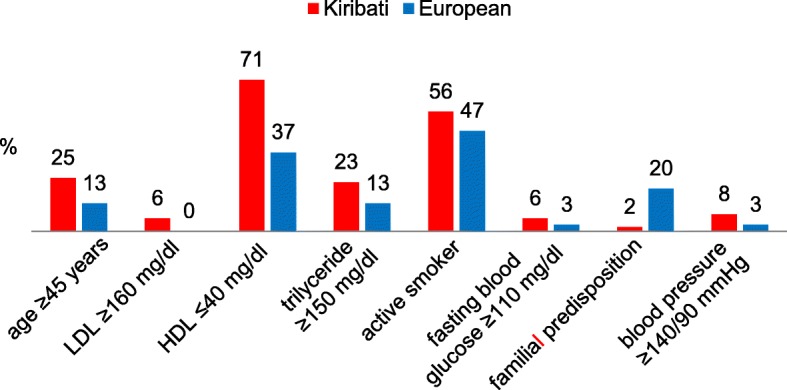
Prevalence (%) of cardiovascular risk factors in Kiribati (*n* = 48) and European (*n* = 29) seafarers

Figure 2 demonstrates the frequency of cardiovascular risk factors in the two different cultural groups. It reveals decreased HDL levels and active smoking as the most frequent risk factors in both groups, especially among the Kiribati, 71% of whom had low HDL values.

### Endurance performance capacity

All conducted tests were age-adjusted and executed up to at least three increasing stress levels. Four subjects (two from each cultural group) were excluded from the exercise test because of taking a heart rate limiting medication or due to knee dysplasia. The subjective fitness evaluation was similar in both cultural groups. All tested subjects reached around 60–80% of their individual maximal heart rates. Both cultural groups displayed similar results within each age group, without significant differences. The average VO_2_ was 39 ml/kg/min in Kiribati crew members and 42 ml/kg/min among Europeans (*p* = 0.087).

### Activity monitoring

The evaluation of activity showed significantly higher total energy expenditure per day among the Kiribati in comparison to the European seafarers. As shown in Table 2, Kiribati walked an average of 2000 steps more than the Europeans (who more frequently performed administrative duties due to their often higher-rank position on board).

**Table 2:** Daily activity profiles according to Bodymedia armband monitors and activity protocols

	Kiribati (*n* = 46)	European (*n* = 30)	*p* ^*1*^
Total energy expenditure (kcal) (SD)	3622 (465)	3354 (583)	0.033
Active energy expenditure (kcal) (SD)	1315 (466)	1142 (583)	n.s.
Daily physical activity (h) (SD)	4.0 (1.7)	3.5 (1.9)	n.s.
Steps (number of) (SD)	14,209 (3716)	12,247 (3027)	0.023
Sleeping hours (h) (SD)	5.2 (1.1)	5.8 (1.0)	0.038
Working time (h) (SD)	9.5 (1.4)	10.2 (1.2)	n.s.

^1^Mann-Whitney-U-Test

The analysis of working time according to the daily protocols revealed an average of approx. 10 h a day for both groups, corresponding with a working week of 70 h. The seafarers had a low average sleep duration with 5.5 h a day.

According to the Perceived Stress Scale, Kiribati were more often assigned to the higher stress-risk group (total sum of 20 points or more) than Europeans (15.2% vs. 3.2%; *p* = n.s.).

### Lifestyle parameters

The sailors were interviewed on their nutritional behaviour on land and aboard the current cargo vessel. Kiribati often claimed to eat significantly larger amounts of food aboard, while most European sailors stated that they ate less or about the same during their shipboard stay [28].

Concerning cigarette consumption, the Kiribati were more often active smokers than the Europeans (56.3% vs. 47.2%; *p* = n.s.). When including former smokers, the cigarette consumption of Kiribati seafarers was 17.6 pack years (as opposed to 10.4 pack years among Europeans). Significant differences were noted concerning the alcohol consumption − 19.1% of the Kiribati claimed to be completely abstinent, while only 3.0% of Europeans stated that they did not drink alcohol (*p* = 0.011).

In respect of physical activity for strengthening the cardiovascular system, more than 50% of the sailors did not engage in regular physical activities while ashore or at home. During their stay aboard the cargo vessels, an additional decrease in physical activity in relation to the time spent doing active sport on land was observed (Kiribati and Europeans 33 and 43%, respectively; *p* = n.s.).

### Health education

**Fig. 3:**
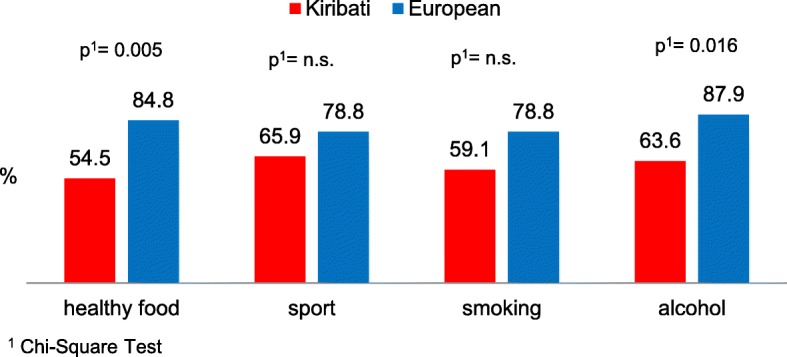
Frequency (%) of Kiribati (*n* = 44) and European (*n* = 33) crew members feeling properly informed on different relevant health issues

The need for intensified health education was also estimated differently between the two groups (Fig. 3). This difference was especially striking concerning knowledge about healthy food: while a total of 84.8% of the Europeans felt well-informed on this issue, only half of the Kiribati stated having an appropriate knowledge.

## Discussion

The evaluation of cardiovascular risks in large population studies (e.g. the PROCAM or Framingham study) usually relies on homogenous (German or American) collectives. Therefore, the application of the respective established risk scores was not suitable for the present multicultural population. In this study, the summation of the eight internationally established cardiovascular risk factors revealed that 28.2% of the sailors had 3 or more risk factors. Compared to the study results of Oldenburg et al. (2008) (with a percentage of 34.2% within a collective of 161 sailors) [23], this number is relatively low. Considering only the Kiribati seafarers, 35.4% had at least three risk factors corresponding to the results of the study mentioned above. Apart from smoking habits, the values most often found were lower HDL and higher triglyceride values and, with that, big differences in non-HDL cholesterol.

In the total group, 68.8% of the sailors had a BMI ≥ 25 kg/m^2^. This data confirms the results of previous maritime studies describing elevated weight in 66% of examined seafarers [29]. Also older studies displayed similar results for certain population groups on cargo vessels, where 51.5% of the European crew members were overweight [6]. This value even lies below average according to the OECD (2012) [13], where a comparison between eastern and western Europeans shows a percentage of 55.0 and 61.3% respectively.

Among the Kiribati, 91.7% were classified as overweight. This is also not surprising in view of their larger waist circumference, which correlates highly with cardiovascular risks due to visceral fat distribution [30]. It is necessary to compare the BMI levels of the Kiribati seafarers with the data of the non-seafaring Kiribati ashore (Table 3). Unfortunately, the available data is not stratified according to age groups, but demonstrates at least a general overview of the male adult population [31].

**Table 3:** BMI of examined Kiribati seamen and the average male Kiribati population ashore (≥ 20 years of age)

	Examined Kiribati aboard ship (*n* = 48)	Kiribati population ashore
BMI ≥ 25 kg/m^2^, (%)	91.7%	76.5%
BMI ≥ 30 kg/m^2^, (%)	45.9%	39.3%

Although Kiribati is already one of the countries worldwide with the highest prevalence of obesity [32], the investigated group aboard had even higher values than the reference population ashore. This result is especially alarming as the examined seafarers belong to an occupational group with high physical efforts. Their relatively high level of physical activity corresponds with their high work-related calorie expenditure and thus their verifiably good cardiac fitness level.

The fact that the examined Kiribati subjects between 20 and 29 years in this study were of almost normal weight (with a BMI averaging at 26 kg/m^2^) suggests that Kiribati seafarers usually are not overweight at the beginning of their career. These values contrast those of non-seafaring young Kiribati ashore with an obesity rate (BMI ≥ 30 kg/m^2^) of 47.2% [12]. This difference is probably caused by a training effect, as the Kiribati sailors have to attend training over a period of at least 18 months in a maritime training centre before their first employment on board. The regular physical training in this centre seems to lead to an almost normal weight among sailors, especially in comparison to the non-seafaring Kiribati population within the same age group.

The weight information (from prior medical fitness tests) proves that Kiribati have a massive gain in weight during the course of their stay aboard the vessels. Many sailors stated that they do less sport during their stay aboard in comparison to the time on land. This supports the development of adiposity and thereby increases the risk of cardiovascular diseases. Due to the distinct increase in weight among Kiribati, it is necessary to place an emphasis on nutritional education, particularly as almost half of the Kiribati (45.5%) claimed to require information on this issue (Europeans 15.2%).

Another crucial cardiovascular risk factor on board is smoking. Among the seafarers, 51% were active smokers. This corresponds with other contemporary studies on sailors’ smoking habits [33]. According to statistics from the last couple of years, the percentage of smokers among sailors seems to be decreasing. In the review by Pougnet et al. (2013) [6], 61.3% of sailors examined in the 1990s were classified as active smokers, while in the 2000s only a percentage of 45.4% active smokers was found. The present study documented a larger percentage of active and heavier smokers among the Kiribati crew members in comparison to the Europeans. Cigarettes are still available at very low prices aboard cargo vessels and pose one of the legal intoxicants on board beside alcohol. Monotonous work, loneliness and insufficient health education on board are probable causes for the high prevalence of active smokers among Kiribati.

The documented energy expenditure with an average of 3500 kcal confirms the results of elderly studies by Zorn (1984) [34] and Kierst (1966) [35], in which a sailor working on cargo vessels consumes around 3000 to 3500 kcal when working at average intensity. This is interesting as both comparative studies were conducted at a time when seafaring was regarded to be much more physically demanding than today.

The comparison of the two cultural groups shows that Kiribati have a higher active energy consumption level than European seafarers. This is explainable by the fact that Kiribati, who work only within lower-ranked positions, usually perform more strenuous physical tasks than officers, who are mostly Europeans.

The whole study sample has long working hours on average. The average sleep periods were alarmingly short, especially among the Kiribati crew members. Correspondingly, the Perceived Stress Scale highlights that (particularly the Kiribati) sailors are exposed to an elevated mental stress level, which also represents a cardiovascular risk factor.

At the beginning of the seafarers’ career an education in a Kiribati maritime centre has to be completed successfully to prepare the students for the work and live on board. According to the present findings, the Kiribati should be advised during this maritime education also in a healthy lifestyle. Apart from nutritional counselling [8], more physical exercises, anti-drug campaigns and stress prevention are important aspects that can lead to lowering the risk factors for cardiovascular diseases among sailors.

The relatively small number of subjects represents a limitation of this study. On the other hand the participation rate in the present study was very good. Furthermore, the study was limited by examining the crew of only one company; this may limit the external validity of the study. In light of the relatively low number of Kiribati seafarers examined this study can be regarded as a pilot study. Although it is to be feared that it will be hardly possible to find vessels with such high numbers of Kiribati on board, future studies are welcomed with larger populations of Kiribati. In respect of Kiribati seafarers there is a particular need for intervention studies based on the present findings.

Another limitation is the lack of reference values for the Kiribati population. Therefore, culturally specific characteristics could not be considered when comparing the collectives. Thus, further scientific studies concerning the nutritional behaviour and related cardiovascular risks among different cultures of sailors are necessary.

Finally, we cannot exclude the possibility that the reported differences in cardiovascular risk and corresponding nutrition-related parameters are at least in part due to the existing differences in educational level or job position between Kiribati and European seafarers, since cultural background and educational level and job position are inevitably confounded in a seafarer population. This underlines the need for future intervention studies to ascertain that changes in life-style behaviour of Kiribati result in changes of cardiovascular risk.

## Conclusions

The present results suggest a higher cardiovascular risk among Kiribati seafarers, with nutritionally related factors (obesity and non-HDL cholesterol) seemingly playing a key role. According to WHO fact sheet No. 394 (2015), a precise dietary change could reduce health risks, such as that for cardiovascular diseases. Therefore, the results of the study confirm the necessity of health-promoting interventions aboard cargo vessels. Preventive medical care is apparently required in order to educate seafarers in a number of health issues.

## Data Availability

The datasets used and/or analysed during the current study are available from the corresponding author on reasonable request.

## Abbreviations

BMI: Body-Mass-Index
cm: centimeter
dl: deciliter
DRKS: German Clinical Trials Register
e.g.: exempli gratia (Latin phrase meaning “for example”)
et al.: et alii (Latin phrase meaning “and others”)
Fig.: figure
g: gram
GmbH: Limited liability company
h: hour
HDL: High density lipoprotein
kcal: large calorie
kg: kilogram
LDL: Low-density lipoproteins
m^2^: square meter
mg: milligram
min.: minute
ml: milliliter
mmHg: millimeters of mercury
n.s.: not specified
No: Number
RF: Risk factors
SD: Standard deviation
Tab.: Table
VO2: Uptake of oxygen
vs: versus
WHO: World Health Organization
